# Muscle–Brain crosstalk in cognitive impairment

**DOI:** 10.3389/fnagi.2023.1221653

**Published:** 2023-07-27

**Authors:** Xiaowei Han, Muhammad Ashraf, Srinivas M. Tipparaju, Wanling Xuan

**Affiliations:** Department of Pharmaceutical Sciences, USF Health Taneja College of Pharmacy, University of South Florida, Tampa, FL, United States

**Keywords:** skeletal muscle, sarcopenia, cognitive impairment, Alzheimer's disease, aging

## Abstract

Sarcopenia is an age-related, involuntary loss of skeletal muscle mass and strength. Alzheimer's disease (AD) is the most common cause of dementia in elderly adults. To date, no effective cures for sarcopenia and AD are available. Physical and cognitive impairments are two major causes of disability in the elderly population, which severely decrease their quality of life and increase their economic burden. Clinically, sarcopenia is strongly associated with AD. However, the underlying factors for this association remain unknown. Mechanistic studies on muscle**–**brain crosstalk during cognitive impairment might shed light on new insights and novel therapeutic approaches for combating cognitive decline and AD. In this review, we summarize the latest studies emphasizing the association between sarcopenia and cognitive impairment. The underlying mechanisms involved in muscle**–**brain crosstalk and the potential implications of such crosstalk are discussed. Finally, future directions for drug development to improve age-related cognitive impairment and AD-related cognitive dysfunction are also explored.

## 1. Introduction

Aging is often considered an inevitable progressive process along with deteriorating functional decline. With increased longevity and decreased mortality, population aging has been sweeping the world rapidly in recent years (Beard et al., 2016). In the United States, there were more than 56 million individuals aged over 65 years in 2020, which accounts for 16.9% of the national population, and is estimated to be roughly 22% by 2050.^1^

Aging is often associated with decreased function in multiple key organs, including the brain, skeletal muscle, and the heart (North and Sinclair, 2012; Hambright et al., 2019; Hou et al., 2019). The strategy connecting this triple functional system could be a key to Alzheimer's disease (AD) prevention and rehabilitation. Physical and cognitive impairments are two major causes of disability in the elderly. Sarcopenia, an age-related involuntary loss of skeletal muscle mass and strength, is strongly associated with AD, a neurodegenerative disease with a prevalence of over 47 million globally (DeTure and Dickson, 2019; Beeri et al., 2021). Evidence from both clinical (Salinas-Rodriguez et al., 2021; Hu et al., 2022; Ramoo et al., 2022) and animal studies (Nagase and Tohda, 2021; Lee and Lim, 2022) demonstrates that skeletal muscle dysfunction may be a key factor that can contribute to cognitive impairment. So far, there is no effective cure for sarcopenia or AD. Drugs approved for Alzheimer's disease are classified into acetylcholinesterase inhibitors and N-methyl-D-aspartate receptor antagonists (Lin et al., 2021). However, AD patients cannot benefit much from these treatments due to the biological restriction of the blood-brain barrier (BBB), the low bioavailability, poor pharmacokinetics and pharmacodynamics of these drugs (Nunes et al., 2022). Current therapies or drugs available for AD focus on managing symptoms and mostly target the acetylcholinesterase system, which so far has turned out to generate mild effects with low clinical outcomes (Marucci et al., 2021). In addition, currently, there are no drugs approved by the Food and Drug Administration for the treatment of sarcopenia (Cho et al., 2022).

Drug treatments provided for both Alzheimer's Disease and sarcopenia remain ineffective; however, exercise has been proven to be highly effective in maintaining muscle mass and cognitive health (Beckwee et al., 2019; Huang et al., 2022). A recent study showed that exercise enhanced muscle-derived circulating factors release, which increased synaptic plasticity and hippocampal neurogenesis and thus improved cognitive function via muscle–brain crosstalk (Rendeiro and Rhodes, 2018). Therefore, early diagnosis and intervention for sarcopenia may benefit AD.

Based on earlier studies, in the present review, we summarize the latest findings on the association between sarcopenia and cognitive impairment. We also discuss the underlying mechanisms responsible for muscle–brain crosstalk. Finally, we will explore the potential strategies for targeting such crosstalk and future directions for drug development to improve cognitive function during aging and AD.

## 2. Association between sarcopenia and cognitive impairment

Skeletal muscle is the largest organ in the human body by weight and is responsible for maintaining body posture and performing voluntary movements (Tieland et al., 2018). Normal physiological functioning of skeletal muscle allows both physical activity and metabolic regulation. Thus, changes in skeletal muscle function and mass may significantly affect metabolism due to its sensitivity to insulin (Stump et al., 2006). Interestingly, the brain is also an insulin-sensitive metabolic organ that consumes 25% of the glucose in the body (Rossi et al., 2001). Therefore, there is potential endocrine crosstalk between skeletal muscle and the brain. Both animal studies and clinical trials have shown a strong association between sarcopenia and cognitive impairment. It has been demonstrated that skeletal muscle atrophy may have detrimental effects on cognitive function in multiple animal models. For instance, in 5XFAD transgenic mice (an Alzheimer's disease mouse model), muscle atrophy accelerated the onset of cognitive impairment, and the underlying mechanism may be mediated by hemopexin secreted from the atrophy muscle (Nagase and Tohda, 2021). Myokines released by atrophying muscles caused aberrant energy metabolism and thus impaired cognition in a type 2 diabetes mellitus mouse model (Lee and Lim, 2022). Consistent with these animal studies, clinical studies and meta-analyses revealed that sarcopenia is implicated in increased risk for cognitive impairment (Wu et al., 2021; Hu et al., 2022; Li et al., 2022). A meta-analysis including 18,788 participants based on 26 cohort, cross-sectional, and case-control studies found that participants with sarcopenia showed a higher risk of developing cognitive impairment [OR = 1.75; 95% CI = 1.57, 1.95; *P* < 0.00001]. Additionally, the MMSE score was lower in the sarcopenia group than that in the non-sarcopenia group [OR = −2.23; 95% CI = −2.48, −1.99; *P* < 0.00001] (Chen et al., 2022). Similarly, other meta-analyses demonstrated that sarcopenia is an independent risk factor for cognitive impairment (Chang et al., 2016; Cipolli et al., 2019; Peng et al., 2020). Two recent clinical studies further demonstrated the higher prevalence of cognitive impairment in older adults with sarcopenia. Longitudinal associations between sarcopenia and mild cognitive impairment (OR = 1.74; 95% CI 1.02, 2.96; *P* = 0.04), decreased cognitive function (β = −0.57; 95% CI−0.93, −0.21; *P* < 0.01), immediate verbal recall (β = −0.14; 95% CI−0.28, −0.01; *P* = 0.04), delayed verbal recall (β = −0.12; 95% CI−0.23, −0.01; *P* = 0.03), and semantic verbal fluency (β = −0.17; 95% CI−0.28, −0.05; *P* = 0.01) have been found in a study including 496 older Mexican adults. Sarcopenic elderly adults showed a 0.7% higher annual rate of mild cognitive impairment (Salinas-Rodriguez et al., 2021). Another cohort study including 1,946 respondents in rural Malaysia showed similar results. Sarcopenic elderly adults have an 80% higher risk of cognitive impairment compared with those without sarcopenia (RR 1.80; 95% CI 1.18–2.75) (Ramoo et al., 2022). Taken together, these studies support a strong link between muscle atrophy and cognitive impairment. Early diagnosis and intervention for sarcopenia may impede the progression of cognitive impairment.

## 3. Potential mechanisms of skeletal muscle–brain crosstalk in cognitive function regulation

Clinical studies support a potential association between skeletal muscle and cognitive function, but the underlying mechanisms remain unknown. In this review, we have focused on the association between sarcopenia and cognitive function during aging and AD. It is very likely that the aging brain could have an impact on skeletal muscle function, and it is a challenge to identify all the connecting dots. People with cognitive dysfunction or AD may have less physical activity, causing a decline in muscle function. Skeletal muscle could release different cytokines and other muscle fiber-derived peptides or myokines under distinct conditions (So et al., 2014). However, here, we emphasize the myokines signal within the muscle, which potentially leads to crosstalk between skeletal muscle and the brain, involving age as a common denominator between cognitive dysfunction and AD-related cognitive impairment. Thus, skeletal muscle could act as an active endocrine organ and regulate the function of distant organs or tissues. These cytokines and myokines serve as messengers for communication between skeletal muscle and the brain (Kim et al., 2019). In the present review, we summarize potential beneficial factors and detrimental factors in muscle–brain crosstalk during aging.

### 3.1. Beneficial factors governing muscle and cognitive function

#### 3.1.1. IGF-1

Insulin-like growth factor-1 (IGF-1) is a 70-amino acid polypeptide and is synthesized by hepatocytes and other organs, including skeletal muscle (Yakar et al., 2018; Li et al., 2019). IGF-1 is essential to skeletal myogenesis, which plays a critical role in maintaining muscle mass and function (Vitale et al., 2019). Decreased IGF-1 was observed in muscle atrophy (Grounds, 2002) and was also noted to be essential for brain function. Previous studies demonstrated that IGF-1 deficiency induced cognitive impairment during aging both in human and rodent models (Deak and Sonntag, 2012; Toth et al., 2022). The level of IGF-1 is significantly decreased in sarcopenia patients, which might be due to physical inactivity (Widajanti et al., 2022). Skeletal muscle release of IGF-1 was also decreased in aged mice. However, no specific effects on muscle recovery were observed when IGF-1 alone was replenished; the combination of IGF-1 and exercise was demonstrated to reduce skeletal muscle wasting to some extent (McMahon et al., 2014). This study indicates the specific role of skeletal muscle-secreted IGF-1 in improving muscle loss and cognitive function. So far, no direct evidence shows that exogenous IGF-1 could improve cognitive function during aging. But exogenous supplementation of IGF-1 showed improvement in cognitive function. The beneficial effect is likely due to inhibition of inflammation and oxidative stress (Wang et al., 2020).

#### 3.1.2. Brain-derived neurotrophic factor

Brain-derived neurotrophic factor (BDNF), which is classified into the neurotrophin family, was initially found to be essential for brain development and the nervous system (Chao et al., 2006). BDNF is extensively expressed in the nervous system, but recent studies show that skeletal muscle can also release BDNF, which supports the myokine role of BDNF (Lebrun et al., 2006; Raschke et al., 2013). BDNF also promotes myoblast differentiation, maintains the survival of motor neurons (Sakuma et al., 2015), and functions as a contractile-inducible protein (Moreira-Pais et al., 2022). Therefore, deterioration of skeletal muscle mass in sarcopenia, along with limited physical activity or a sedentary lifestyle in the elderly, may contribute to decreased levels of BDNF. Low BDNF levels were closely related to cognitive impairment, which may increase the incidence of AD (Bathina and Das, 2015; Siuda et al., 2017). Taken together, these findings support the idea that dysregulated BDNF could be the potential link between sarcopenia and AD.

#### 3.1.3. Irisin

In recent years, Irisin has emerged as a key factor secreted mostly from the skeletal muscle that provides beneficial effects to both the skeletal muscle and the brain. At the molecular level, Irisin is a pro-myogenic factor that is a cleaved form of fibronectin type III domain-containing protein 5 (FNDC5) (Moreno-Navarrete et al., 2013). Irisin has been proven to enhance insulin sensitivity and boost glucose and lipid metabolism in skeletal muscle (Shen et al., 2022). Irisin has been demonstrated to ameliorate muscle wasting by accelerating myoblast fusion and protein synthesis (Huh et al., 2014). A recent study found that the level of irisin decreased during aging, and chronic administration of irisin can improve metabolic dysfunction and ameliorate skeletal muscle atrophy in aged mice (Guo et al., 2023). Regarding brain function, the genetic deletion of irisin impaired cognitive function (Islam et al., 2021). As a mediator of muscle-brain crosstalk, irisin improved cognitive function with an increase in BDNF expression (Peng and Wu, 2022). Loss of irisin is involved in cognitive impairment during aging, and exogenous administration of irisin improved cognitive function in the AD preclinical model (Madhu et al., 2022). Hence, irisin may be a promising cure for aging-related sarcopenia and cognitive impairment (Gao et al., 2013).

#### 3.1.4. Secreted protein acidic and rich in cysteine

Secreted protein acidic and rich in cysteine (SPARC) is a novel secretory matricellular glycoprotein, defined as a myokine, which is released by skeletal muscle contraction during exercise (Aoi et al., 2013). SPARC is involved in skeletal muscle biology, which is upregulated during muscle regeneration (Petersson et al., 2013). It could counteract the abnormal deposition and accumulation of adipose tissue in aged skeletal muscle (Ko et al., 2016). In addition, during aging, the level of SPARC is decreased and SPARC knockout mice showed the sarcopenia phenotype (Ghanemi et al., 2022). Overall, there are potential beneficial roles for SPARC in skeletal muscle, and studies demonstrate that SPARC can be a potential new therapeutic target via muscle–brain crosstalk.

### 3.2. Mediators with dual roles

#### 3.2.1. IL-15

Interleukin-15 (IL-15) is a pleiotropic myokine released by skeletal muscle during exercise. IL-15 is widely involved in skeletal muscle metabolism (Quinn et al., 1995) and protects proteins from degradation while improving insulin sensitivity and promoting myogenesis (O'Leary et al., 2017; Nadeau and Aguer, 2019). During aging, the level of IL-15 in skeletal muscle decreases (Quinn et al., 2010) as shown in a cross-sectional study with 160 outpatient elderly people which demonstrated the inverse correlation between plasma IL-15 levels and sarcopenia (Yalcin et al., 2018). The human data correlate well with the animal data, in which it was noted that IL-15 was decreased in gastrocnemius muscle in aged rats (Marzetti et al., 2009). Another study identified that IL-15 could also serve as a detrimental pro-inflammatory factor in the brain and showed increased serum IL-15 levels, which can be utilized as a biomarker for Alzheimer's disease since IL-15 has been extensively studied in AD pathophysiology (Rentzos et al., 2006; Bishnoi et al., 2015). The contradictory roles of IL-15 in the skeletal muscle and in the brain may be due to an aging-related inflammation environment and different sources of IL-15. Low-grade inflammation during aging increases the release of the pro-inflammatory IL-15 from reticular stromal cells and other myeloid cell types, while it decreases the release of IL-15 from skeletal muscle (Naismith and Pangrazzi, 2019). The level of IL-15 could be a potential contributor to exercise benefits in sarcopenia and cognitive function improvement (Tsai et al., 2019; Pahlavani, 2022).

#### 3.2.2. LIF

Leukemia inhibitory factor (LIF) is an important member of the IL-6 type cytokine family with 180 amino acid residues (Nicola and Babon, 2015). Skeletal muscle produces and releases LIF (Broholm and Pedersen, 2010), which is associated with the skeletal muscle homeostasis. Previous reports showed that LIF increased muscle glucose uptake via the PI3K-Akt signaling pathway (Brandt et al., 2015). Additionally, LIF stimulates the proliferation of satellite cells and muscle regeneration, which is a promising therapy for muscle atrophy (Kurek et al., 1998; Broholm et al., 2011). The level of LIF has been found to decline in sarcopenic obesity (Pahlavani, 2022). Importantly, LIF could cross the blood-spinal cord barrier and behave as a neuropoietic cytokine in the central nervous system. LIF is essential for the recovery of the nervous system from injury. Furthermore, LIF mediates inflammatory reactions in AD (Lemke et al., 1996). In AD patients, a higher expression of LIF was observed in degenerating human brains compared with normal brains (Soilu-Hanninen et al., 2010). However, the specific mechanism by which LIF could influence the AD brain remains unknown.

#### 3.2.3. IL-6

Interleukin-6 (IL-6), a member of the cytokine family, has both inflammatory and anti-inflammatory effects. The proinflammatory function of IL-6 is involved in aging-related diseases. Studies in the elderly have found that an increased level of IL-6 is associated with the occurrence of sarcopenia (Bian et al., 2017; Rong et al., 2018). However, enhanced release of IL-6 during muscle contraction also induced myogenic differentiation (Steyn et al., 2019). A combination of recombinant IL-6 and treadmill training in old mice could enhance their endurance training adaptation together with functional capacity improvement (Leuchtmann et al., 2022). However, due to the sedentary lifestyle of sarcopenia patients, the beneficial role of IL-6 may decrease, and the inflammatory effects may overwhelm the anti-inflammatory roles. As an inflammatory factor, IL-6 can cross the BBB and impair brain function (Banks et al., 1994). Inflammatory IL-6 is involved in cognitive impairment during AD. An increase in IL-6 levels in AD brains was observed, and its neutralization or inhibition of the IL-6 signaling pathway alleviated cognition decline (Silva et al., 2021). Based on the above evidence, it is likely that during aging, the release of muscle-derived anti-inflammatory IL-6 decreases, accelerating the progression of AD.

#### 3.2.4. Lactate

Lactate is a metabolic substrate, secreted from skeletal muscle during mechanical muscle contractile stimulation. A previous study showed that lactate promoted myoblast differentiation *in vitro* via myogenic determination protein-dependent signaling pathway, and moreover, lactate could cross the BBB, facilitating the expression of BDNF in the brain (El Hayek et al., 2019). The role of lactate in cognitive function is not clear, and additional studies are necessary for understanding its role in the brain. A cross-sectional study including 2,523 participants showed that a higher plasma lactate level was associated with systemic inflammation and an increased probability of mild cognitive impairment (Pan et al., 2019). However, another study evaluating the cerebrospinal fluid (CSF) lactate in 267 outpatients reported the opposite results, and the level of lactate in CSF was decreased in patients with AD (Bonomi et al., 2021). Therefore, additional studies are needed to explain these contradictory observations.

### 3.3. Detrimental factors governing muscle and cognitive function

#### 3.3.1. Cathepsin B

Cathepsin B is a typical member of the cysteine lysosomal protease family. It is recognized as a myokine released from skeletal muscle following exercise (Kim et al., 2019). An *in vitro* study showed that cathepsin B participated in myotube formation (Jane et al., 2002). During aging, cathepsin B levels were upregulated in microglia, which contributed to the generation of mitochondrial-derived reactive oxygen species (ROS), causing increased inflammation and thereby impaired memory (Ni et al., 2019). Similarly, enhanced translocation of cathepsin B reduced sirtuins and promoted proinflammatory reactions in senescent microglia, resulting in cognitive impairment (Meng et al., 2020). On the other hand, cathepsin B could cross the BBB and promote BDNF expression in the hippocampal area and improve memory function (Moon et al., 2016). It is obvious from the above reports that additional studies are needed to determine the function of cathepsin B in muscle–brain crosstalk in elderly adults.

#### 3.3.2. Myostatin

Myostatin, which is known as growth and differentiation factor 8, is secreted by skeletal muscle. It is a negative mediator in skeletal muscle growth (Gao et al., 2013), which decreases muscle size and mass. Myostatin deficiency is beneficial to skeletal muscle metabolism (Cleasby et al., 2016). Myostatin inhibits the expression of myogenic differentiation-related genes, such as Myod and Myf5, in a smad3-dependent manner (Langley et al., 2002). Myostatin could accelerate proteolysis in the soleus and impede protein turnover *in vivo* and in C2C12 cells. The potential mechanism may be mediated by the phosphorylation of Smad3 (Manfredi et al., 2017). Interestingly, the level of myostatin in 12-month-old double transgenic amyloid precursor protein and presenilin 1 (APP/PS1) mice was elevated, which may trigger skeletal muscle atrophy and cognitive deficits. Knockdown of myostatin with shRNA in these mice attenuated skeletal muscle degradation and memory loss (Lin et al., 2019). Importantly, increased release of myostatin by skeletal muscle in sarcopenia patients promoted cognition decline in the elderly population, thus increasing the risk of AD (Siriett et al., 2006; Bergen et al., 2015).

#### 3.3.3. Growth differentiation factor-15

Growth differentiation factor-15 (GDF-15) belongs to the transforming growth factor β (TGF-β) superfamily. Stress enhances its release. GDF-15 has been reported as a biomarker for sarcopenia. An elevated level of GDF-15 during aging was found, which was related to the decline of skeletal muscle mass and function (Kim et al., 2020). Cross-sectional and 2 year prospective analyses involving 788 participants supported the finding that an increased level of GDF-15 was associated with the prevalent sarcopenia (Kim et al., 2022). GDF-15 has also been recognized as a biomarker for aging-related cognitive decline (Jiang et al., 2016). A cohort study with 1,603 participants demonstrated an association between elevated plasma GDF-15 and an increased risk of dementia (McGrath et al., 2020). Another longitudinal Sydney Memory and Aging Study, consisting of 1,037 participants, also reached similar conclusions (Fuchs et al., 2013). Hence, GDF-15 is a detrimental mediator for muscle–brain crosstalk and a potential target for the treatment of sarcopenia and cognitive dysfunction.

#### 3.3.4. IL-8

Interleukin 8 (IL-8) is a CXC member of the chemokine family, which is a myokine released by skeletal muscle during exercise (Akerstrom et al., 2005). IL-8 acts as a pro-inflammatory factor in sarcopenia (da Costa Teixeira et al., 2023). A UK cohort study including 336 community-dwelling elderly men and women demonstrated that an elevated IL-8 level was associated with an increased risk of sarcopenia (Oflazoglu et al., 2020). Consistent with this, several clinical studies have shown that sarcopenia in the elderly had higher levels of IL-8 compared with the non-sarcopenia group (Fan et al., 2022; Teixeira et al., 2022). Interestingly, IL-8 is also identified as a biomarker during AD progression (Swardfager et al., 2010; Alsadany et al., 2013). In addition, a higher level of IL-8 in the elderly was associated with poorer cognitive performance (Baune et al., 2008). The potential mechanism may be related to its role in microglia migration toward Aβ deposits associated with senile plaques and activation of microglial cells (Li et al., 2009). However, besides skeletal muscle cells, other cell types, including macrophages and endothelial cells, also release IL-8 (Nielsen and Pedersen, 2007; Luo et al., 2022). Therefore, the cellular sources of IL-8 and its biological role in sarcopenia and AD need further study.

## 4. Targeting the skeletal muscle to combat cognitive decline

### 4.1. Exercise

Different studies support the fact that exercises benefit the skeletal muscle system and improve memory function. Here, we summarize aerobic exercise and resistance exercise, which are two major types of exercise, along with their effects on skeletal muscle and cognitive function during aging.

#### 4.1.1. Aerobic exercise

Multiple animal studies demonstrate the beneficial effects of AE on sarcopenia. These studies show that AE improved skeletal muscle atrophy in sarcopenia mice and reversed chronic inflammation and dysfunctional mitochondria via sestrin2 in an AMPK alpha-2-dependent manner (Liu et al., 2021). Lifelong aerobic exercise activates autophagy and inhibits protein degradation via AMPK/PGC-1α signaling, thereby improving aging-related muscle atrophy (Liang et al., 2021). Similar findings showed the therapeutic effects of habitual aerobic exercise on sarcopenia in a senescence-accelerated mice prone8 model via enhanced mitochondrial maintenance and muscle protein synthesis (Aoki et al., 2020). Despite promising results in animal experiments, so far limited clinical studies regarding AE and its beneficial effects in humans are inconclusive. A cross-sectional study including older women treated with aerobic training, secondary lifestyle, and resistance training showed that AE could not decrease the prevalence of sarcopenia, but resistance training was effective (Supriya et al., 2022). AE showed beneficial effects on cognitive impairment as well. A meta-analysis involving 1,364 mild cognitive impairment participants demonstrated that AE could improve the cognitive function of older adults with mild cognitive impairment (Yong et al., 2021). The underlying mechanism of such beneficial effects might be due to activation of the NF-κB/miR-503/BDNF pathway (Niu et al., 2018), increased myokines (BDNF and IGF-1), and reduction of inflammatory cytokines (Tsai et al., 2018, 2019).

#### 4.1.2. Resistance exercise

Resistance exercise (RE) is a form of exercise intended to increase muscular strength and endurance. Consistent evidence from clinical trials supports the benefits of RE in sarcopenia. RE improves muscle strength, muscle quality, and muscle performance in elderly adults with sarcopenia (Chen et al., 2021; Mende et al., 2022; Zhao et al., 2022). Potential mechanisms include the rejuvenation of satellite cells (Hsu et al., 2022) and the improvement of mitochondrial and autophagic function in skeletal muscle (White et al., 2016). Furthermore, a number of multilevel meta-analyses were used to demonstrate that RE enhances cognitive function regardless of cognitive status and age (Northey et al., 2018; Wilke et al., 2019; Landrigan et al., 2020; Zhang et al., 2020). It is believed that myokines are the key factors in RE that contribute to cognitive function improvement. However, it was demonstrated that RE could either increase or demonstrate no effect on the IGF-1 level (Titus et al., 2021). Furthermore, compared with traditional resistance exercise, the combination of RE and cognitive tasks improved brain function and BDNF level (Castano et al., 2022). The effect of RE on myokine release was also sex-dependent, and previous studies showed that mixed low-resistance training only increased plasma levels of BDNF in male participants, but no changes in female participants were noted. RE could be beneficial to counteract sarcopenia and memory loss in elderly adults. However, RE might be suitable only for early intervention. Patients suffering from dementia, AD, or sarcopenia are significantly associated with a low level of physical activity, higher disability, and poor quality of life. Thus, physical exercise is not a good option for these patients. In either case, a better understanding of the molecular and cellular mechanisms that mediate the benefits of physical activity will help in the development of potential therapeutic approaches.

#### 4.1.3. Muscle-targeted strategies for cognitive function improvement

Compared with the brain, muscles have more accessibility for intervention, especially using invasive strategies. Preclinical studies have shown that several muscle-targeted treatments enhance cognitive function. Muscle-specific overexpression of neprilysin and scFv59 or knockdown of myostatin using genetic approaches showed favorable effects on the brain. Overexpression of neprilysin in the muscle reduces Aβ amyloid deposits in the brain (Li et al., 2020), while increased scFv59 expression in the muscle reduces Aβ amyloid levels in the cerebrospinal fluid (Yang et al., 2013). Cell-based therapy is another promising strategy. For example, intramuscular injection of stem cells releasing regenerative factors enhanced neurogenesis and astrogliogenesis in the aged mouse hippocampus (Huntsman et al., 2018). These muscle-targeted strategies have translational potential in cognitive impairment therapy.

## 5. Limitations and perspectives

**Figure 1:**
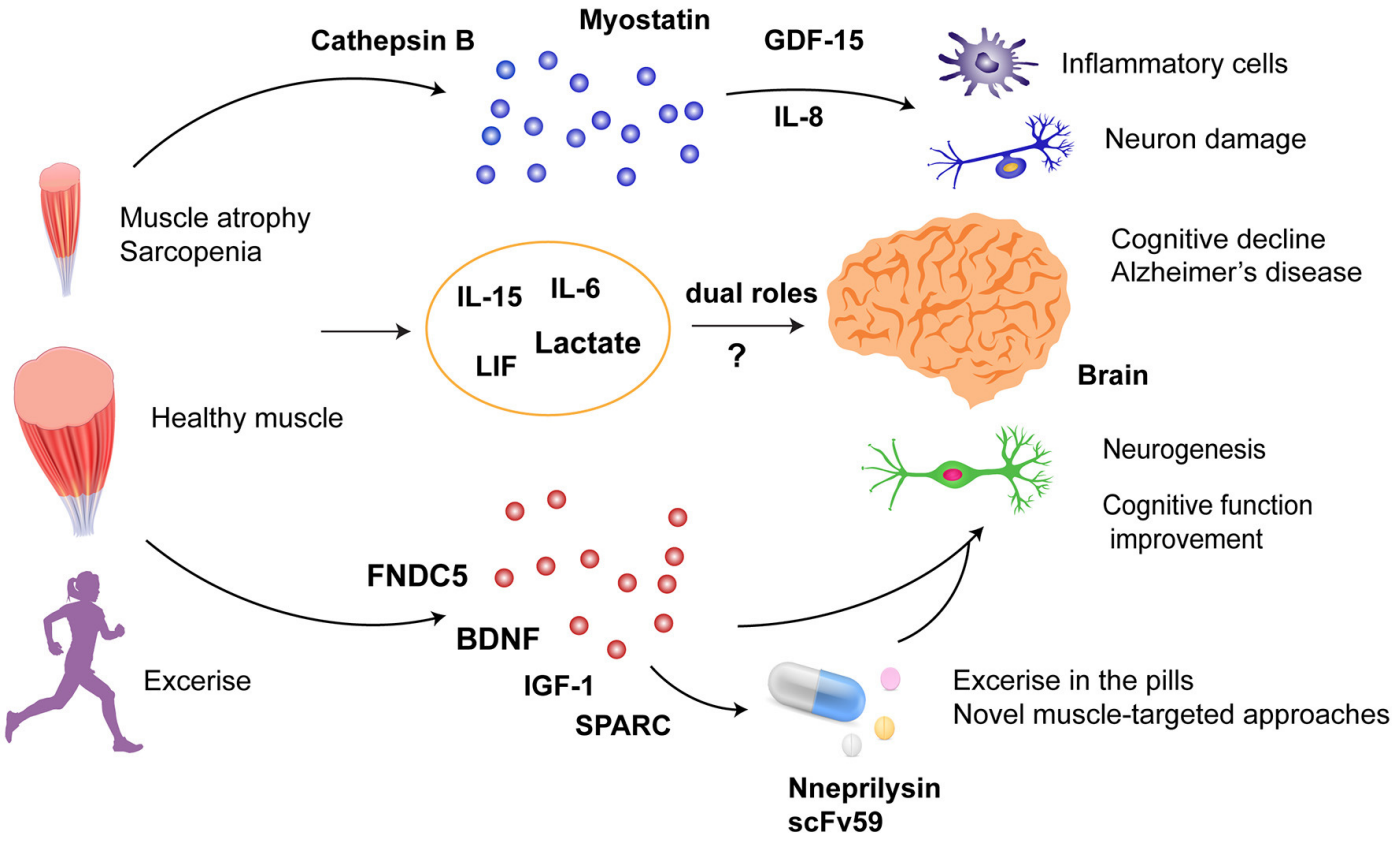
Muscle–brain crosstalk in cognitive dysfunction. Myokines or cytokines released from skeletal muscle affect neurons and inflammation in the brain: (1) detrimental factors: cathepsin B, myostatin, and GDF-15; (2) dual roles: IL-15, IL-6, LIF, and lactate; (3) beneficial factors: FNDC5, BDNF, IGF-1, and SPARC. Exercise-enhancing release of beneficial factors and specific overexpression of neprilysin and scFv59 in the skeletal muscle could be promising strategies against cognitive dysfunction. GDF15, Growth differentiation factor-15; IL-8, interleukin 8; IL-15, interleukin-15; IL-6, interleukin-6; LIF, leukemia inhibitory factor; FNDC5, fibronectin type III domain containing protein 5; BDNF, brain-derived neurotrophic factor; IGF-1, insulin-like growth factor-1; SPARC, secreted protein acidic and rich in cysteine.

Sarcopenia and dementia are common geriatric diseases. AD is the most common cause of dementia and the fifth leading cause of death in elderly adults. Importantly, the estimated total healthcare costs for the treatment of AD in 2020 were estimated at US $305 billion, which is expected to increase to more than US $1 trillion as the population ages (Wong, 2020). To date, no effective cures for AD have been reported. Therefore, drug development for cognitive dysfunction and AD is important. Clinical observations and pre-clinical studies revealed muscle–brain crosstalk on cognitive function (Figure 1). In pre-clinical studies, it seems that myokines are the key mediators in muscle–brain crosstalk during cognitive dysfunction, but currently, no clinical trials on the effects of these myokines have been conducted. Exercise seems to be a promising intervention for sarcopenia and cognitive impairment. Due to physical inactivity in patients with sarcopenia or AD, exercise might not be the first-line intervention for patients in the late stages of the disease. However, exercise could be used as a platform to discover potential beneficial factors contributing to a favorable outcome. In addition, muscle-specific conditional knockout animals and AD preclinical models are useful for studying the underlying mechanisms. A few muscle-targeted approaches via the regulation of muscle gene expression, extrinsic supplementation, and stem cell transplantation showed promising results to improve cognitive function or promote neurogenesis. However, for future clinical applications, dose-dependent efficacy, pharmacokinetics, and delivery routes need to be taken into consideration.

## 6. Conclusion

Evidence from pre-clinical studies and clinical observations supports the idea that muscle–brain crosstalk plays a critical role in cognitive function. Muscle-targeted intervention is promising for improving aging or AD-related cognitive decline. In the present review, we outlined three principal areas for skeletal muscle and brain crosstalk, namely, (a) beneficial strategies, (b) mediators that play dual roles (i.e., protective and damaging roles), and (c) strategies that may cause increased risk and advance the disease condition during aging.

## Author contributions

XH drafted the manuscript. ST, MA, and WX reviewed and edited the manuscript. All authors have read and approved the final manuscript.

## Footnotes

^1^ https://www.americashealthrankings.org/explore/senior/measure/pct_65plus/state/ALL
